# Joint Optimization of Energy Consumption and Data Transmission in Smart Body Area Networks

**DOI:** 10.3390/s22229023

**Published:** 2022-11-21

**Authors:** Limiao Li, Junyao Long, Wei Zhou, Alireza Jolfaei, Mohammad Sayad Haghighi

**Affiliations:** 1 School of Computer Engineering and Applied Mathematics, Changsha University, Changsha 410219, China; 2 School of Software Engineering, Central South University, Changsha 410017, China; 3 School of Software and Electrical Engineering, Swinburne University of Technology, Hawthorn, VIC 3122, Australia; 4 College of Science and Engineering, Flinders University, Bedford Park, SA 5042, Australia; 5 School of Electrical and Computer Engineering, College of Engineering, University of Tehran, Tehran 1417935840, Iran

**Keywords:** smart city, body area networks, edge computing, energy optimization

## Abstract

In Wireless Body Area Networks (BAN), energy consumption, energy harvesting, and data communication are the three most important issues. In this paper, we develop an optimal allocation algorithm (OAA) for sensor devices, which are carried by or implanted in human body, harvest energy from their surroundings, and are powered by batteries. Based on the optimal allocation algorithm that uses a two-timescale Lyapunov optimization approach, we design a framework for joint optimization of network service cost and network utility to study energy, communication, and allocation management at the network edge. Then, we formulate the utility maximization problem of network service cost management based on the framework. Specifically, we use OAA, which does not require prior knowledge of energy harvesting to decompose the problem into three subproblems: battery management, data collection amount control and transmission energy consumption control. We solve these through OAA to achieve three main goals: (1) balancing the cost of energy consumption and the cost of data transmission on the premise of minimizing the service cost of the devices; (2) keeping the balance of energy consumption and energy collection under the condition of stable queue; and (3) maximizing network utility of the device. The simulation results show that the proposed algorithm can actually optimize the network performance.

## 1. Introduction

**Figure 1:**
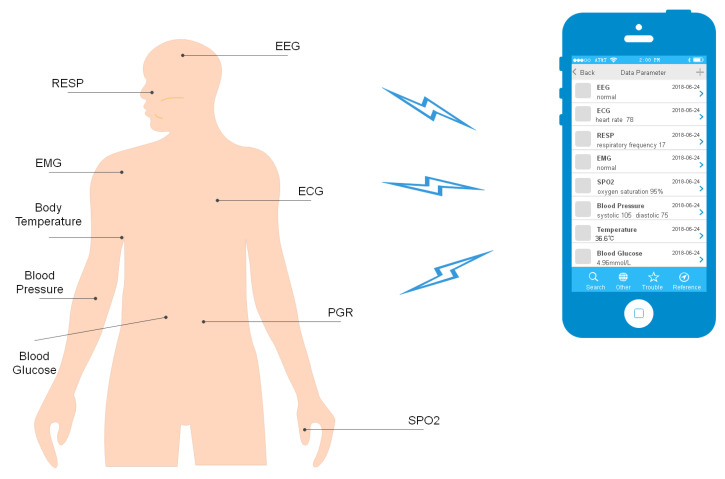
Common sensor node locations in BAN.

Wireless sensor networks have always been the core technology of the process industry in the framework of Internet of Things. Their first bold appearance was in personal area networks for which many standards have been developed [1]. Now, they are widely used in the healthcare industry too. Medical expenses and limited resources are always the key issues [2], and that is why body area networks (BAN) have attracted great attention. They are an extension to wireless sensor network technology, while playing an important role in the Internet of Things architecture. According to the United Nations data on aging, it is estimated that by 2050, the proportion of elderly people in the total population will reach 21%. Chronic diseases are diseases that consume the most medical resources and require constant monitoring. As it happens, the elderly are the most vulnerable to chronic diseases. Therefore, as the trend of population aging increases, the focus of researchers in the global medical community is mainly on monitoring systems [3]. Efforts are being made to turn Internet of Things into Internet of Everything, including BAN. BAN is a wireless network formed by physiological parameter collection sensors on the human body or biosensors transplanted into the human body [4]. It is worth noting that these sensors’ power consumption is low. They monitor the condition of the body and collect physiological information, such as respiratory signals (RESP), electrocardio signals (ECG) [5], blood pressure, blood glucose, etc., and finally, they transmit the collected data to the monitoring equipment safely. Compared with the data collected in hospital for examination and analysis, BAN is obviously more convenient and effective, reducing the consumption of medical resources as well as medical costs. However, BAN still has some problems that need to be addressed. Figure 1 shows the locations where sensor nodes collect information in the body area network.

Energy resource limitation is a major issue in BANs. This is a well-known problem in sensor networks and its derivatives that keeps bouncing back in different scenarios [6,7,8,9]. Optimizing the management of energy is a step towards sustainability [10], which can extend the capabilities of wireless sensors to provide biomedical services. Generally, the sensor is powered by a battery. Since these sensors are wearable or implanted in the human environment, their sizes and weights cannot be too large, which implies that the battery capacity is unlikely to be large. The battery of wearable sensors can be replaced, but it is a problem when it comes to implantable sensors, because most implantable sensors do not allow battery replacement, and many important physiological parameters are collected by sensors implanted into the human body. If the sensor is only powered by the battery, as the battery power decreases, the physiological information monitored by the sensor will be affected or even deviated, which will seriously affect the quality results of BAN. Under these conditions, many researchers brainstormed and developed energy harvesting (EH) technology [11]. Sensors have modules that use EH to collect different forms of energy from the surrounding environment and convert it into electricity for data collection, storage, and transmission.

Data communication is another major issue in BAN. When wireless sensors collect human physiological information as biomedical monitoring, the low-quality (incomplete or non-real-time) data communication is very likely to cause human life and safety to be threatened. Therefore, data communication is very sensitive in BAN. Although cognitive radio (CR) technology [12] has been proposed to improve the quality of data communication, and it does enhance the stability of data communication, it does not solve the problem completely. Data communication problems are related to real time and integrity [13]. Real-time performance is linked to data delay, and integrity involves data collection efficiency and transmission efficiency. In many cases, we are tolerant of monitoring data, and allow for some delay as long as it does not fall beyond a certain range. However, we are very strict about the efficiency of data collection and transmission, since incomplete or missing data will seriously interfere with BAN and may even endanger human lives. In order to enable BAN to provide efficient and safe services for patients, data communication, especially data collection rate and transmission rate, has top priority.

Admittedly, EH technology and CR technology have made great contributions to BAN, but there are still some issues that need to be further addressed. It is advantageous that we do not need to know the specific process of energy collection, however, since energy collection is a dynamic and random process, we should make greater demands on energy consumption. Cognitive radio and its extension effectively solve the problem of data delay, so we must focus on data integrity. Under these challenges, we consider that a single sensor device’s system, operating in a frame consisting of multiple time slots, joins the sleep/wake time modes. Sleep/wake decisions have been used in many areas, such as video analytics [14]. In theory, the eternal awake state is only a perfection of the hypothesis, but in practice, the system switches between sleep and wakefulness [15]. In each frame, the system selects to go into the sleep mode or to stay awake. In the wake state, data monitoring, data collection, storage and transmission operate normally. If the node chooses to go into the sleep mode, it will not respond to any requests except for energy collection. Compared with the previous BAN environment, we add a new sleep operation to help deal with energy problems. First, we mainly conduct biomedical monitoring for patients with chronic diseases. In other words, for the vast majority of patients, we only need periodic monitoring to obtain reliable data, since redundant monitoring will waste power. Second, no matter how high the energy collection efficiency is, the collected energy will never be greater than the energy consumed, so adding a sleep mode to the system can alleviate the battery’s power load.

In this paper, we study the optimal allocation of resources for a single sensor in BAN by jointly optimizing the energy and data of the sensor [16]. We propose an optimal allocation algorithm (OAA) that combines the two-timescale Lyapunov optimization approach. We use the Lyapunov optimization approach in OAA to optimize the joint framework for forming network service cost and network utility, and decompose it into three stochastic processes, namely battery management, data collection control, and transmission energy control. The OAA is then used to solve the problem of minimizing network service costs and maximizing network utility while capturing randomly generated energy harvesting processes and ensuring the stability of the BAN system. The main contributions of this paper are summarized as follows:

- We adopt the sleep/wake two-timescale model to join the WBAN system, adding an accessible sleep state when the node is not transmitting data, and only collecting energy without energy consumption. This two-timescale model, combined with weight perturbation, is applied to the Lyapunov technique to “push” energy to a non-zero value to avoid energy faults, which provides the possibility of sustainable power supply;
- We utilize the two-timescale Lyapunov optimization technique and decompose the problem into battery management, data collection and quantity control, and transmission energy consumption control. We also design an optimal allocation algorithm (OAA) of low complexity, which updates queue dynamically, and ensures network utility and service cost optimization at each time slot;
- We introduce a network service cost that combines energy consumption and data transmission at the same time, and the optimized network service cost is equivalent to optimizing energy consumption and data transmission at the same time. We also analyze and simulate the performance of the proposed OAA. The results show that the proposed algorithm can optimize the network performance in reality.

The remainder of the paper is organized as follows. In Section 2, related works are reviewed. In Section 3, we describe the system model. In Section 4, we propose an OAA and present the proposed framework, and then analyze the stability and performance of the proposed algorithm in Section 5. In Section 6, simulation results are provided to evaluate the performance of OAA. In Section 7, we conclude the paper and shed light on some future works.

## 2. Related Work

As a wireless sensor network integrated with biomedicine, BAN has its own particularity. The most important aspect of research on BAN is to improve the requirement of energy and data problems.

In WBAN, research on energy harvesting and energy efficiency has been conducted to improve the performance of systems. In [17], Yang et al. propose to use an antenna which is composed of corrugated metal–insulator–metal plasmonic structures, the triple-band rectenna for low power application can convert the harvested radio frequency power into dc power to improve energy harvesting. In [18], Qi et al. propose an adaptive TDMA-based protocol which can be dynamically adjusted to maintain the harvested energy amount by link scheduling. This protocol is a promising candidate for realizing the lifetime action in EH-WBANs. In [19], Gurinderjeet Kaur Natt and Rajanpreet Bhatti design a hybrid protocol named Body Area Network Enhanced Critical Heterogeneous Adaptive Threshold Sensitive Stable Election Protocol(BAN E-CHATSEP). The protocol modifies the transmission criteria and calculates the current sensing values for every biomedical sensors on the basis of their respective real world medical data. This protocol is energy efficient and improves performance. In [20], the authors present a clockless pipeline compressed sensing encoder for highly energy-efficient purpose. The addition of Quasi-Delay-Insensitive circuits improves the sub-threshold robustness, and the zero value detector is added to reduce power consumption. In [21], Amit Samanta et al. propose a network management cost minimization framework to solve the network throughput and energy management under the constraint of the QoS. However, these system models could not reflect the data problem very well, although the energy optimization has been fulfilled.

Through the expansion of CR technology, there are also many methods designed to solve data problems. In [22], Tanmoy Maitra et al. propose a load distributed MAC protocol, which distributes the load of data transmission among the nodes in each posture, and obtains an optimal scheme to increase the data transmission rate. In [23], Peng et al. propose that devices use a cooperative multiple input multiple output (MIMO) technique in relay cooperative strategy and operate as the cooperative MIMO in direct cooperative strategy. This method can reduce the average bit error ratio to save energy and improve data transmission. In related research work [8,9,10], the system model is optimized by Lyapunov to obtain dynamic and accurate data. In [24], the authors use Lyapunov optimization to maximize the expected good bits per packet transmission for the source node in wireless communication system. At the same time, battery levels and bit error rates are bounded to ensure data transmission. In [25], Qiu et al. focus on decode-and-forward-based cooperative wireless communications with an EH relay and optimize the long-term average symbol error rate based on the Lyapunov optimization theory. Then, the proposed strategy analyzes the corresponding diversity order and EH gain, and the result is a reduction in the bit error rate and an increase in the data transfer rate. The proposed algorithm can achieve much better performance than existing methods based on Markov decision process and water-filling in [24,25]. In [26], Huang develops an optimal sleep/wake scheduling algorithm that combines energy and data optimization without prior knowledge of EH.

In WBAN, energy management and data interaction are inseparable. In order to improve system performance, they both must be studied at the same time. The weight between them must also be balanced to maximize system performance. To fill this research gap, this paper proposes a framework to capture the stochastic process of energy harvesting and sleep/wake mode switching, and designs an optimal allocation algorithm which can minimize the network service cost and maximize network utility. At the same time, this algorithm can jointly optimize energy and data, and ensure the weight balance between energy and data. As described in [27], data transmission not only needs to pay attention to speed, but it also needs to pay attention to privacy. Compared with [27], although our algorithm cannot achieve such high privacy protection, the transmission capacity and endurance capacity are more prominent in the case of good security. In [28], the authors pay more attention to charging delay, but our work focuses on battery sustainability. In [29], Chan Haeng Lee et al. propose a packet scheduling scheme, although the amount of data transmitted is large and the accuracy of data is comparable to our scheme, our algorithm is slightly better in sustainability. The three-layer edge cloud integration framework proposed in [30] has solved the problem of energy consumption and data volume, but it cannot avoid the problem of time delay. We have achieved a good balance in time delay, energy consumption, and data transmission.

## 3. System Model Design

We design a system that consists of a single sensor device (called the node in the following text), i.e., the node collects data in the human body and transmits data through channel to the destination server. The system can provide a statistics function and communication service to the device user and destination server. The node is powered by a battery and can receive energy from the surrounding environment. As an extension of the sensor network, the body area network also runs periodically. Time is slotted and is divided into frames of size T, i.e., t∈T={0,1,2,⋯}.

### 3.1. Sleep/Wake Model and the Channel State

In each frame *m*, the node can choose to enter the wake or sleep state. We model the sleep/wake decisions with $SW(m_{t})$, where $m_{t}=\left\lfloor t/T\right\rfloor$. Specifically, $SW(m_{t})=1$ if the node remains awake during frame *m*. Otherwise the node goes to sleep, expressed as $SW(m_{t})=0$.

If the node is in the wake state in frame *m*, we consider transmitting data to the destination server through the channel. We use S(t) to represent the channel state of the node within time slot *t*, wherein S(t)=1 means the channel is idle and can normally transmit data, while S(t)=0 means the channel is busy and cannot transmit data to the destination server. In the following, we assume that S(t) is i.i.d. in every time slot and let $\pi_{s}=Pr\left\{S\left(t\right)=1\right\}$ for simplicity.

### 3.2. The Data Collection and Transmission Model

In a frame where the node remains in wake mode, the node can receive data. We use G(t) to denote the amount of new data received in time slot *t*, and the upper limit $G_{max}$ of the amount of collected data of node at a time slot is given. Additionally, the range of G(t) is:

(1) $$0\leq G\left(t\right)\leq G_{max}$$

if the node is in sleep mode during frame time, then G(t)=0.

Network utility is determined by the amount of data collected by the node, which is expressed as U(G(t)). The utility function U(G(t)) is assumed to be continuously derivable and differentiable, which increases with the increase of the amount of the node’s collected data G(t). In addition, the utility function U(G(t)) is concave under the bounded first derivative, and U(0)=0. We use ψ to denote the maximum first derivative of the utility function U(G(t)), i.e., $\psi =U^{\prime }\left(0\right)$.

As a behavior of data collection, the node can also transmit data in frames that maintain wake mode. Under the influence of channel state S(t) and transmission distribution energy consumption $P_{\mu }\left(t\right)$, the function $\mu \left(t\right)=\mu (S\left(t\right),P_{\mu }\left(t\right))$ denotes the amount of data transmitted by node through the channel in time slot *t*.

We also define a channel capacity β(t) and assume that β(t) is i.i.d. across different time slots. The maximum capacity of the channel is $\beta_{max}$, and the data transmission amount μ(t) is constrained by the channel capacity:

(2) $$0\leq \mu \left(t\right)\leq \beta \left(t\right)\leq \beta_{max}$$

### 3.3. Data Queue Dynamic Model

We use Q(t) to denote the data queue of the node. The input of the data queue is the collected data amount G(t), while the output is the data amount μ(t) transmitted by the node to the destination server. The dynamic change of the data queue is:

(3) $$Q\left(t+1\right)=Q\left(t\right)-SW\left(m_{t}\right)\mu \left(t\right)+SW\left(m_{t}\right)G\left(t\right)$$

with Q(0)=0. In addition, in time slot *t*, the amount of data that a node can transmit should not exceed the amount of data in the data queue, so the following limitation applies:

(4) 0≤μ(t)≤Q(t)

### 3.4. Energy Consumption Model

When the node collects or transmits data, it will consume energy. There is a linear relationship between the energy consumption caused by data collection and the amount of data collected. We use $P_{G}G\left(t\right)$ to denote the energy consumption of data collection, wherein $P_{G}$ denotes the energy consumption per unit of data. We use $P_{\mu }\left(t\right)$ to denote the energy consumption used to distribute the transmission data, and that every feasible transmission energy consumption satisfies the constraint $P_{min}^{\mu }\leq P_{\mu }\leq P_{max}^{\mu }$ for some $P_{min}>0$ and $P_{max}<\infty$. Constraining $P_{min}^{\mu }$ here is to illustrate that compared to the sleep state, the awake state consumes more energy in order to monitor the capture node, even if it simply stays idle and does nothing.

We used two time scale models of sleep/wake, so there was also energy consumption for the two-state transitions. However, the conversion energy is very small and not every time slot will undergo state conversion. In this paper, we will not consider the conversion energy consumption for the time being. Therefore, the total energy consumption of node in time slot *t* is:

(5) $$P_{total}\left(t\right)=P_{G}G\left(t\right)+P_{\mu }\left(t\right)$$

Since the amount of data collected $G\left(t\right)<G_{max}$, and the energy consumption allocated for data transmission $P_{\mu }\leq P_{max}^{\mu }$, it can be seen that the maximum energy consumption of any node in a time slot is:

(6) $$P_{max}=P_{G}G_{max}+P_{max}^{\mu }$$

### 3.5. Energy Collection Model and Energy Queue Dynamic Model

The nodes can receive energy from their surrounding environment. The harvestable energy in time slot *t* is represented by *A*, that is, the maximum harvestable energy to nodes in time slot *t*. Assuming $h_{max}<\infty$, for all time slots *t*, we obtain:

(7) $$0\leq h\left(t\right)\leq h_{max}$$

Next, for convenience, we assume the energy queue has infinite capacity, and that the node can decide whether or not to harvest energy in each time slot. We define a variable by using r(t)∈[0,h(t)] to denote the amount of energy actually harvested by the node at time slot *t*. It is important to note that the energy collection behavior is not affected by the sleep/wake state of the node.

Similar to the data queue, E(t) denotes the energy queue of the node. The dynamic change of the energy queue is:

(8) $$E\left(t+1\right)=E\left(t\right)-SW\left(m_{t}\right)P_{total}\left(t\right)+r\left(t\right)$$

with E(0)=0. It is obvious that the energy consumed by the node in time slot *t* should not exceed the energy available in the energy queue, which is called the energy availability constraint:

(9) $$0\leq P_{total}\left(t\right)\leq E\left(t\right)$$

In the current situation, by using the queuing dynamic of energy, we start by assuming that the energy queue has an infinite capacity. As we will show later, our algorithm guarantees a deterministic energy storage boundary.

### 3.6. Utility Maximization of Network Service Cost Management

According to the system model described above, we model the utility maximization problem of network service cost management. This model is divided into two parts, one is to optimize the management of network service cost, i.e., to minimize the cost, and the other part is to maximize network utility U(G(t)). Next, we will explain the first part, network service cost, in detail.

At present, the major challenges are energy and data problems, so we set up the network service cost to realize the joint optimization of energy and data. On the one hand, we study the energy consumption of data transmission, if there is a transmission energy consumption, which incurs energy consumption cost $a_{E}$ per joule, and our objective is to minimize the energy consumption cost to solve the energy problem. On the other hand, we also analyze quantities of data, if the data are transmitted through function μ(t), which induces data transmission cost $a_{Q}$, to minimize the cost of data transmission and to maximize transmission efficiency. Thus, it is suitable and desirable to minimize both types of costs in order to optimize the system.

We will use the network service cost [31] as the performance index, i.e., the weighted transmission data cost is subtracted after the transmission energy cost is weighted. Our goal is to minimize the cost of redundancy between these, which is the cost of network services. We define network service cost N(t) in the time slot *t* as:

(10) $$N\left(t\right)=w_{E}a_{E}P_{\mu }\left(t\right)-w_{Q}a_{Q}\mu \left(t\right)$$

wherein, the first term presents the energy cost, the second term stands for the data cost, and $w_{E}$ and $w_{Q}$ are the weights of the transmission energy consumption cost and transmission data cost, respectively.

When $w_{E}\gg w_{Q}$, the network is more sensitive to energy problems. When $w_{Q}\gg w_{E}$, the network places more emphasis on data problems. We assume that $a_{E}$, $a_{Q}$, $w_{E}$, and $w_{Q}$ are all positive and bounded.

**Remark 1.**  *Since each time slot channel transmission is assumed to be independent and identically distributed. In this paper, in order to guarantee fairness to energy and data, for all time slot t, we set the weight of transmission energy consumption cost and transmission data cost to be the same, i.e., $w_{E}=w_{Q}$.*

In cases where the channel transmission are not statistically identical, the weights can be adjusted accordingly, and the proposed Lyapunov optimization method is still applicable.

We define function f(t) as the utility maximization function of network service cost management, which is expressed as:

(11) $$f\left(t\right)=U\left(SW\left(m_{t}\right)G\left(t\right)\right)-SW\left(m_{t}\right)N\left(t\right)$$

When the node enters sleep mode, it cannot interact with the data, then f(t)=0.

## 4. The Optimal Allocation Algorithm

In this section, we introduce our algorithm, i.e., the best algorithm to solve problems, which is based on Lyapunov theory and combined with weight perturbation. The network service cost management utility maximization problem is decomposed into three sub problems in a single time slot, and the optimization framework is described in detail.

### 4.1. Problem Decomposition Based on Lyapunov Optimization

In time slot *t*, the state of the body area network consists of the data queue Q(t) and the energy queue E(t) of the node, which is expressed as M(t)=(Q(t),E(t)). Now, we select a perturbation value *O* (to be specified later). Then, a perturbed Lyapunov function is defined as:

(12) $$L\left(t\right)=\frac{1}{2}{\left[Q\left(t\right)\right]}^{2}+\frac{1}{2}{\left[E\left(t\right)-O\right]}^{2}$$

The purpose of using *O* here is to keep the Lyapunov function value small, and we make *E*(*t*) values approach *O*. Therefore, by seriously choosing the value of *O*, we can guarantee that the energy queue always has enough energy when the node is awake.

L(t) is a scalar measure. After that, we use $\hat{E}\left(t\right)=O-E\left(t\right)$ to denote the residual energy capacity of the node. The higher the available energy, the lower the residual energy capacity. A Lyapunov drift $\Delta_{T}\left(t\right)$ [32] is defined under the condition of body area network state M(t):

(13) $$\Delta_{T}\left(t\right)=E\left\{L(t+T)-L(t)|M(t)\right\}$$

This expectation is taken over energy acquisition, data collection, and energy distribution, the randomness in choosing the sleep/wake decisions, and the randomness of the channel state at each time slot.

In each time slot, we try to minimize Lyapunov drift $\Delta_{T}\left(t\right)$, which can cause the data queue Q(t) to reach the stable state, at the same time, the residual energy capacity will also be narrowed, and the node will receive energy from the surroundings as much as possible. Please note that the goal of the utility maximization problem of network service cost management is to optimize the network service cost and the utility of the data collected by the body area network. In order to achieve this goal, we add the utility maximization function of network service cost management to Lyapunov drift $\Delta_{T}\left(t\right)$ and construct the drift-plus-utility function $\Delta_{T,V}\left(t\right)$:

(14) $$\Delta_{T,V}\left(t\right)=\Delta_{T}\left(t\right)-\sum_{t=0}^{T-1}E\left\{Vf\left(t\right)|M\left(t\right)\right\}$$

wherein *V* is a non-negative parameter, and is used to control the algorithm performance, indicating the proportion of function f(t) in $\Delta_{T,V}\left(t\right)$. The lower the value *V*, the lower the proportion of f(t) in $\Delta_{T,V}\left(t\right)$, and vice versa. By minimizing $\Delta_{T,V}\left(t\right)$, we can jointly stabilize queue length and optimize function f(t).

**Lemma 1.**  *Under any feasible sleep/wake decision, data collection behavior, transmission energy distribution action that satisfies the energy availability constraint, and energy harvesting behavior that can be implemented at any time slot t, the upper limit of $\Delta_{T,V}\left(t\right)$ is:*

(15) $$\begin{array}{cc} \Delta_{T,V}\left(t\right)\leq & BT+\sum_{t=0}^{T-1}E\left\{-\hat{E}\left(t\right)r\left(t\right)|M\left(t\right)\right\}\\ + & \sum_{t=0}^{T-1}E\left\{Q\left(t\right)SW\left(m_{t}\right)G\left(t\right)\right.\\ + & \hat{E}\left(t\right)SW\left(m_{t}\right)P_{G}G\left(t\right)\\ - & \left.VU\left(SW\left(m_{t}\right)G\left(t\right)\right)|M\left(t\right)\right\}\\ - & SW\left(m_{t}\right)\sum_{t=0}^{T-1}E\left\{Q\left(t\right)\mu \left(t\right)-\hat{E}\left(t\right)P_{\mu }\left(t\right)\right.\\ - & \left.V{\tilde{a}}_{E}S\left(t\right)P_{\mu }\left(t\right)+V{\tilde{a}}_{Q}\mu \left(t\right)|M\left(t\right)\right\} \end{array}$$

*wherein ${\tilde{a}}_{E}=a_{E}w_{E}$ and ${\tilde{a}}_{Q}=a_{Q}w_{Q}$. Here, the value of B is determined by the system parameter and is independent of the performance parameter V, which is expressed as:*

(16) $$B=\frac{1}{2}\left(G_{max}^{2}+\beta_{max}^{2}+h_{max}^{2}+P_{max}^{2}\right)$$

Proof is provided in Appendix A.

According to the Lemma, the right side of the formula is the basis of our algorithm design and analysis. The value of *B* is independent of the variable to be optimized. In order to display our algorithm, the following functions are defined:

(17) $$\begin{array}{cc} D_{R}\left(t\right)\; & \leq \sum_{t=0}^{T-1}E\left\{-\hat{E}\left(t\right)r\left(t\right)|M\left(t\right)\right\}\\ \; & +\sum_{t=0}^{T-1}E\{Q\left(t\right)G\left(t\right)+\hat{E}\left(t\right)P_{G}G\left(t\right)\\ \; & -VU(G(t))|M(t)\}\\ \; & -\sum_{t=0}^{T-1}E\{Q\left(t\right)\mu \left(t\right)-\hat{E}\left(t\right)P_{\mu }\left(t\right)\\ \; & -V{\tilde{a}}_{E}S\left(t\right)P_{\mu }\left(t\right)+V{\tilde{a}}_{Q}\mu \left(t\right)\left|M\left(t\right)\right\} \end{array}$$

Please note that $D_{R}\left(t\right)$ roughly represents the controllable components on the right side of the formula when $SW(m_{t})=1$ and S(t)=1.

Therefore, we only need to minimize $D_{R}\left(t\right)$ to obtain the optimal energy acquisition quantity $r^{*}\left(t\right)$, data collection quantity $G^{*}\left(t\right)$, and transmission energy consumption quantity $P_{\mu }^{*}\left(t\right)$. Since $D_{R}\left(t\right)$ only involves the energy acquisition quantity h(t) and other information in the current time slot *t*, the minimization of $D_{R}\left(t\right)$ only depends on the current time slot information.

### 4.2. Joint Optimization Framework Structure of Network Service Cost and Network Utility

It is obvious that Equation (17) consists of three parts linearly. Thus, the minimization of $D_{R}\left(t\right)$ can be decomposed into three sub-problems, namely, battery management problem, data collection amount control problem, and transmission energy consumption control problem. The problem of battery management is to optimize the energy acquisition amount r(t), and the problem of amount of data collection control and the energy consumption control of transmission is to optimize the data collection amount G(t) and transmission energy consumption amount $P_{\mu }\left(t\right)$, respectively. Solving these problems requires only information about the node itself, so the node can solve it in a distributed way. After the three sub-problems are solved, the node updates the length of the data queue and the energy queue to prepare for the optimization of the next time slot.

**Figure 2:**
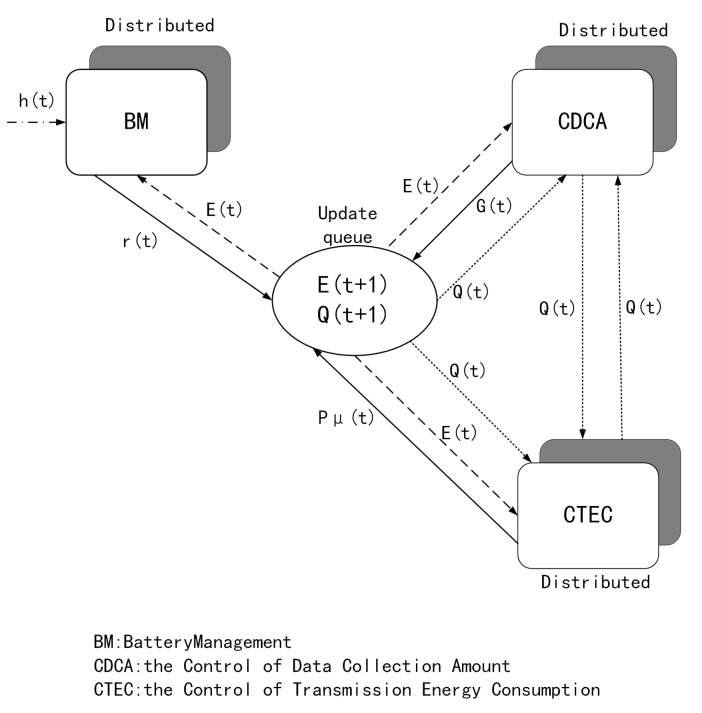
Diagram of three sub problems and data interaction among them.

Figure 2 shows the information required to solve the three sub-problems, the updates to the two queue lengths, and the data interactions between them. Next, we try to analyze and solve the three sub problems in detail.

By observing $\Delta_{T,V}\left(t\right)$, we know that here the expectation is taken over S(t) and $SW(m_{t})$, and the control variables are the data collection amount G(t), the transmission energy consumption amount $P_{\mu }\left(t\right)$ and the quantities of transmission data μ(t). We set the $D_{R}\left(t\right)$ for S(t) and $SW(m_{t})$ to formulate the optimal hypothesis. If the node enters the wake state, the channel is idle, i.e., $SW(m_{t})=1$, S(t)=1. Otherwise, if the node goes to sleep, it sets $G\left(t\right)=P_{\mu }\left(t\right)=\mu \left(t\right)=0$, or if the channel is busy, it sets $P_{\mu }\left(t\right)=\mu \left(t\right)=0$. Therefore, only when the node enters the wake state and the channel is idle can we normally consider the following three sub-problems:

#### 4.2.1. Sub-Problem 1—Battery Management

Considering the first term on the right of $D_{R}\left(t\right)$ and the previous restrictions, we construct the battery management sub-problem. We want to obtain the energy collection volume r(t) of the node in time slot *t*:

(18) $$\begin{array}{cc} min: & \;-\hat{E}\left(t\right)r\left(t\right)\\ s.t. & \;\left\{\begin{array}{c} 0\leq r(t)\leq h(t)\\ E(t)+r(t)\leq O \end{array}\right. \end{array}$$

Battery management is a linear programming problem. We assume that $r^{*}\left(t\right)$ is the optimal solution to this problem. Please note that during the energy collection process, when the energy is less than *O*, the node will always perform the energy collection behavior, otherwise, it can refuse to collect energy. For all time slots *t*, $E\left(t\right)\leq O+h_{max}$. This is very important because it represents our algorithm can achieve finite energy storage capacity instead of infinite battery capacity, i.e., the battery capacity is $O+h_{max}$. The selection of the perturbation value *O* is important, and it can directly influence whether the energy is collected.

Why we select *O* rather than direct select battery capacity $O+h_{max}$ is worthy of mention—if the battery is long-termly charged to full capacity, its capacity will gradually become smaller, which is not conducive to the target of sustainable supply energy, so we choose such a healthy state value *O*. It can be seen from the above that at the beginning of time slot *t*, the battery collects as much energy as possible E(t)<O, that is, $\hat{E}\left(t\right)>0$, then $r^{*}\left(t\right)=min\left(O-E\left(t\right),h\left(t\right)\right)$, otherwise, the node will not collect energy, i.e., $r^{*}\left(t\right)=0$. In short, the solution of the battery management problem gives $r^{*}\left(t\right)$, and the node will collect as much energy as possible to reach the healthy state value *O* of the battery capacity.

In practice, our algorithm generally does not refuse to collect energy unless the battery capacity is full. This shows that the energy we actually store will always be no less than the energy under the algorithm. Therefore, all the behaviors under the algorithm are valid.

#### 4.2.2. Sub-Problem 2—The Control of Data Collection Amount

Considering the second term on the right of $D_{R}\left(t\right)$ and the previous restrictions, we construct the sub-problem of the control of data collection amount. We want to obtain the data collection amount G(t) of the node in time slot *t*:

(19) $$\begin{array}{cc} min: & \;G\left(t\right)\left[Q\left(t\right)+P_{G}\hat{E}\left(t\right)\right]-VU\left(G\left(t\right)\right)\\ s.t. & \;0\leq G\left(t\right)\leq G_{max} \end{array}$$

The utility function U(G(t)) is a concave function and is derivable, so our data collection amount control problem is a convex optimization problem. Assuming that $G^{*}\left(t\right)$ is the optimal solution to this problem, based on the convex optimization theory, it can be seen that:

(20) $$G^{*}\left(t\right)={\left[U{{}^{\prime }}^{-1}\left(\frac{Q\left(t\right)+\hat{E}\left(t\right)P_{G}}{V}\right)\right]}_{0}^{G_{max}}$$

where ${\left[x\right]}_{z}^{y}=min\left(max\left(x,z\right),y\right)$, and $U{{}^{\prime }}^{-1}\left(\cdot \right)$ denotes the first derivative of the inverse function of utility function U(·).

#### 4.2.3. Sub-Problem 3—The Control of Transmission Energy Consumption

Considering the third term on the right of $D_{R}\left(t\right)$ and the previous restrictions, we construct the sub-problem of the control of amount of transmission energy consumption. We want to obtain the transmission energy consumption amount $P_{\mu }\left(t\right)$ of the node in time slot *t*:

(21) $$\begin{array}{cc} min: & \;P_{\mu }\left(t\right)\left(\hat{E}\left(t\right)+V{\tilde{a}}_{E}\right)-Q\left(t\right)\mu \left(t\right)-V{\tilde{a}}_{Q}\mu \left(t\right)\\ s.t. & \;P_{min}^{\mu }\leq P_{\mu }\leq P_{max}^{\mu } \end{array}$$

Rate function μ(t) is also a derivable concave function, so it is also a convex optimization problem of transmission energy consumption control. Compared with the data collection amount control problem, it is more complex. We assume that $P_{\mu }^{*}\left(t\right)$ is the optimal solution to this problem. According to the convex optimization theory£°,

(22) $$P_{\mu }^{*}\left(t\right)={\left[\mu {{}^{\prime }}^{-1}\left(\frac{\hat{E}\left(t\right)+V{\tilde{a}}_{E}}{Q\left(t\right)+V{\tilde{a}}_{Q}}\right)\right]}_{P_{min}^{\mu }}^{P_{max}^{\mu }}$$

where $\mu {{}^{\prime }}^{-1}\left(\cdot \right)$ denotes the first derivative of the inverse function of rate function μ(·).

### 4.3. Implementation of OAA

The OAA algorithm we propose does not require any prior knowledge, which is very advantageous in the case of some statistical difficulties in prior information, where it is very difficult to design a low complexity algorithm. Therefore, our OAA algorithm first uses Lyapunov optimization, and then uses low-complexity resource allocation to obtain the optimal solution.

According to current research, Markov decision or Hungarian algorithm are very popular. The time complexity of the Hungarian algorithm is $O(nkL+L^{2}log\left(min\left(n,k\right)\right))$ [33], and the complexity of the Markov decision [34] is exponentially increasing with the number of nodes. The complexity of our OAA algorithm for resource allocation is linear, which is very suitable for the body area network.

## 5. Performance Analysis of OAA

In this section, we analyze the performance of OAA algorithm. We derive the upper limit of the data queue and energy queue of the node, and prove the stability of the body domain network when implementing the algorithm. Mioreover, according to the upper limit of the length of energy queue (i.e., the battery capacity) we set the healthy state value *O* to support body area network operation. When the energy achieves healthy state, as long as the node can transmit data or receive data, it will definitely have enough energy to support.

### 5.1. The Upper Limit Analysis of Data Queue and Energy Queue

**Theorem 1.**  *In the algorithm, for ψ and O previously defined, and for any non-negative parameter V, we use $Q_{max}$ and $E_{max}$ to denote the maximum length of data queue and energy queue respectively:*

(23) $$\;Q_{max}=V\psi +G_{max}$$

(24) $$E_{max}=O+h_{max}$$

*For the node, the data queue length and energy queue length meet at any time:*

(25) $$0\leq Q\left(t\right)\leq Q_{max}$$

(26) $$0\leq E\left(t\right)\leq E_{max}$$

**Proof.**  First of all, we can clearly know that when time slot t=0, Q(0)=0. The data queue length completely satisfies the boundary constraint of the formula. We use mathematical induction to prove that the boundary constraint holds for any time slot. Now, we assume that this boundary is valid for Q(t), and we want to prove that the boundary is also valid for Q(t+1). If the node collects data with the optimal solution $G^{*}\left(t\right)$ obtained from the sub-problem of data collection quantity control, then $VU^{\prime }\left(G^{*}\left(t\right)\right)=Q\left(t\right)-P_{G}\left(E\left(t\right)-O\right)$. Next, we can consider two situations:

1. When $P_{G}\left(E\left(t\right)-O\right)\leq 0$, we know that $Q\left(t\right)\leq VU^{\prime }\left(G^{*}\left(t\right)\right)$. Since ψ is the upper limit of the first derivative of utility function ‘U(G(t))‘, Q(t)≤Vψ, and the upper limit of data collection is $G_{max}$, we can obtain $Q\left(t+1\right)\leq Q\left(t\right)+G_{max}\leq V\psi +G_{max}$;
2. When $P_{G}\left(E\left(t\right)-O\right)\geq 0$, we know that $Q\left(t\right)\geq VU^{\prime }\left(G^{*}\left(t\right)\right)$, i.e., $V\psi \leq Q\left(t\right)\leq V\psi +G_{max}$. According to the sub-problem constraint of data collection amount control under the algorithm decomposition, we can know that G(t+1)=0, so $Q\left(t+1\right)\leq Q\left(t\right)\leq V\psi +G_{max}$.

At this point, the upper limit of the length of the data queue is proved.

Similarly, we can see that whenever E(t)>O, our algorithm selects r(t+1)=0 in this time slot, so $E\left(t\right)\leq O+h_{max}$ is met in any time slot.

The upper limit of the length of the energy queue is also proved, so Theorem 1 is proved. □

### 5.2. Analysis on Node Battery Capacity Size

We have previously determined that the battery capacity is $O+h_{max}$, so now we only need to focus on the size of the perturbation value *O* (i.e., health status value). In the node of the battery capacity of no less than $O+h_{max}$, namely, health values are not less than the perturbation value *O*, if the energy queue length of the node is less than the maximum energy consumption within a time slot, namely, $E\left(t\right)<P_{max}$, the node will not perform data collection or transfer.

**Theorem 2.**  *Under this algorithm in the body area network, if the perturbation value O satisfies:*

(27) $$O=max\left(\frac{V\psi }{P_{G}},\varsigma V\left(\psi +{\tilde{a}}_{Q}\right)+\varsigma G_{max}-V{\tilde{a}}_{E}\right)+P_{max}$$

*when the energy queue length $E\left(t\right)<P_{max}$, the nodes will not transmit and collect data.*

**Proof.**  The process of data collection and data transmission of the node requires energy consumption. First of all, as long as $E\left(t\right)<P_{max}$, nodes will not collect data. The data collection control sub-problem determines the data collection amount of the node within each time slot. Since the utility function U(G(t)) is concave and $U{{}^{\prime }}^{-1}\left(G\left(t\right)\right)$ is negatively correlated with G(t), we know that the condition that the node will not collect data is:

(28) $$\frac{Q\left(t\right)+P_{G}\hat{E}\left(t\right)}{V}\geq \psi =U^{\prime }\left(0\right)$$

We can substitute $\hat{E}\left(t\right)=O-E\left(t\right)$ into the above equation to obtain:

(29) $$O\geq \frac{V\psi }{P_{G}}+E\left(t\right)$$

In order to meet the constraint that when $E\left(t\right)<P_{max}$, the node cannot collect data, and *O* can be set as:

(30) $$O\geq \frac{V\psi }{P_{G}}+P_{max}$$

Next, as long as $E\left(t\right)<P_{max}$, the node will not consume energy for data transmission. According to the transmission energy control sub-problem, the energy consumed by the node in each time slot for transmitting data is determined. Since the transmission-rate function μ(t) is also a concave function, and $\mu {{}^{\prime }}^{-1}\left(t\right)$ is negatively correlated with $P_{\mu }\left(t\right)$, the condition that the nodes will not consume energy for data transmission is:

(31) $$\frac{\hat{E}\left(t\right)+V{\tilde{a}}_{E}}{Q\left(t\right)+V{\tilde{a}}_{Q}}\geq \varsigma =\mu^{\prime }\left(0\right)$$

We can substitute $\hat{E}\left(t\right)=O-E\left(t\right)$ into the above equation to obtain:

(32) $$O\geq \varsigma \psi V+\varsigma G_{max}+\varsigma V{\tilde{a}}_{Q}-V{\tilde{a}}_{E}+E\left(t\right)$$

In order to meet the constraint that when $E\left(t\right)<P_{max}$, nodes cannot consume energy for data transmission, and *O* can be set as:

(33) $$O\geq \varsigma \psi V+\varsigma G_{max}+\varsigma V{\tilde{a}}_{Q}-V{\tilde{a}}_{E}+P_{max}$$

Combining these two formulas, the proof is completed. □

### 5.3. Optimality of the OAA Algorithm

In this section, we use Theorem 3 to prove that the network utility obtained by our proposed OAA algorithm is not much different from the optimal network utility.

**Theorem 3.**  *We use $\bar{U}$ to represent the average time utility obtained by the OAA algorithm, $U^{*}$ is the optimal average time utility of our problem. The following formula shows the gap between $\bar{U}$ and $U^{*}$:*

(34) $$\bar{U}\geq U^{*}-\frac{B}{V}$$

Proof is provided in Appendix B. From Theorem 3, we can see that the network utility of the OAA algorithm has a very small gap between the network utility and the optimal algorithm. Among them, *B* is a constant, *V* denotes how much the network utility is. Therefore, with the increase of *V*, the OAA algorithm and the gap between the optimal algorithm gets smaller and smaller. In addition, the OAA algorithm is very practical because it does not require any prior knowledge.

## 6. Simulation Results

In this section, we present the simulation results to evaluate the performance of OAA. It is worth mentioning that the implementation of OAA algorithm is through the commercial mathematical software MATLAB, and the experimental results are drawn by the data analysis software ORIGIN. At the beginning, we only designed a single sensor in the model part to facilitate understanding. However, for the accuracy of experimental results, we later consider a WBAN consisting of four sensor nodes. That is, we use four sensor nodes to simulate the actual heart rate sensor, the specification of the heart rate sensor is HKX-08A, the internal voltage is 2.4 to 5, the current is 500 to 800, the external voltage can be 30 to 1000, and the battery capacity is 5 × 10^6^. The basic specifications of each sensor are the same, with the exception that the internal voltage is 0.6 to 1.5, the capacity is 1.5 × 10^6^, and they are executed in parallel.

Similar to [23], we use $U\left(G\left(t\right)\right)=log\left(1+\pi_{s}G\left(t\right)\right)$ as the utility function and define a data transmission function $\mu \left(t\right)=log(1+\pi_{s}\left(P_{\mu }\left(t\right)-P_{min}^{\mu }\right))$ [24]. The maximum values of the first derivative of the utility function and the data transmission function are ψ=1 and ς=1, respectively. We set the patient’s environment to be randomly distributed [0, 2000]. The energy consumption of the node for data sampling is $P_{G}=0.2$, and the maximum data collection is $G_{max}=25$. The maximum harvestable energy is $h_{max}=15$, and the actual energy acquisition of the nodes in the time slot *t* is evenly distributed in [0, $h_{max}$]. The probability that each time slot channel is empty is $\pi_{s}=0.5$, and the probability that the node in the sleep/wake decision is in awake mode in time slot *t* is 0.4. The wake-up probability value of sleep/wake-up mode is strictly designed according to the sunshine of about 10 hours a day. The energy is mainly collected from solar energy and the device can collect energy only in wake-up state. The wake-up/sleep ratio of sunshine time is 2:1, and the wake-up/sleep ratio of other times is 1:2. Therefore, the wake-up probability is theoretically close to the proportion of sunshine time. It is worth mentioning that if the probability of awakening is too low, it may cause accidents, and too high will cause redundancy. The maximum energy consumption of the node for data transmission is $P_{max}^{\mu }=10$, and the minimum value is $P_{min}^{\mu }=1$. The maximum channel capacity is $\beta_{max}=6$. In this experiment, we treat data and energy as being of the same importance, i.e., $w_{E}=w_{Q}=1$. The cost of the data transmission and the energy consumption are normalized, i.e., $a_{Q}=1$ and $a_{E}=1$ per Joule [25]. The specific simulation parameters and values are listed in Table 1 below:

### 6.1. Network Utility and Network Service Cost

**Figure 3:**
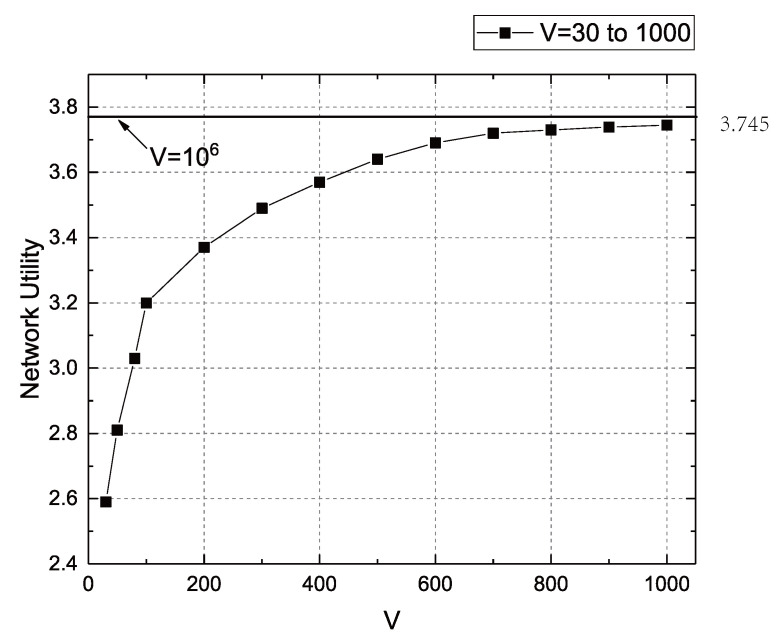
Relationship between *V* and network utility.

Figure 3 shows the network utility when the value of *V* rises from 30 to 1000. From the figure, we can clearly see that the network utility increases with the increase of the *V* value, but the increase rate of the network utility decreases as *V* increases, i.e., the slope decreases. This is consistent with the conclusion of Theorem 3, that is, the network utility function is a concave function. In the experiment, the final network utility reached 3.745 at V=1000. At the same time, we take the network utility at $V=10^{6}$ as the optimal solution to compare the network utility at V=1000, in order to verify the optimality of network utility. It can be seen from the figure that the effect of *V* increasing from 1200 to $10^{6}$ on network utility is far less than the impact of *V* increasing from 30 to 1200 on network utility. Therefore, we can conclude that the network utility at V=1000 is almost the optimal solution for network utility.

**Figure 4:**
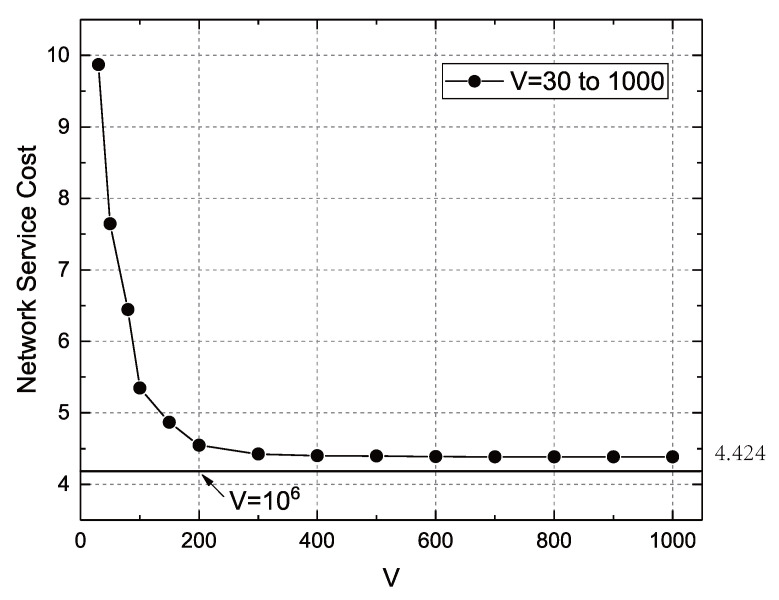
Relationship between *V* and network service cost.

Figure 4 shows the network service cost when the value of *V* rises from 30 to 1000. As can be seen from the figure, when *V* increases, the network service cost gradually decreases, and the rate of reduction in network service costs decreases as *V* increases. This is also in line with Theorem 3, that is, the data transmission function is a concave function of the *V* value. In our experiments, the network service cost dropped to 4.424 at V=300, and then *V* increasing to 1000 or even $10^{6}$ has a very limited impact on network service cost, which also verified the optimality of network service cost. We know that the network service cost at V=300 is close to the optimal solution.

### 6.2. Dynamic Changes in the Queue

**Figure 5:**
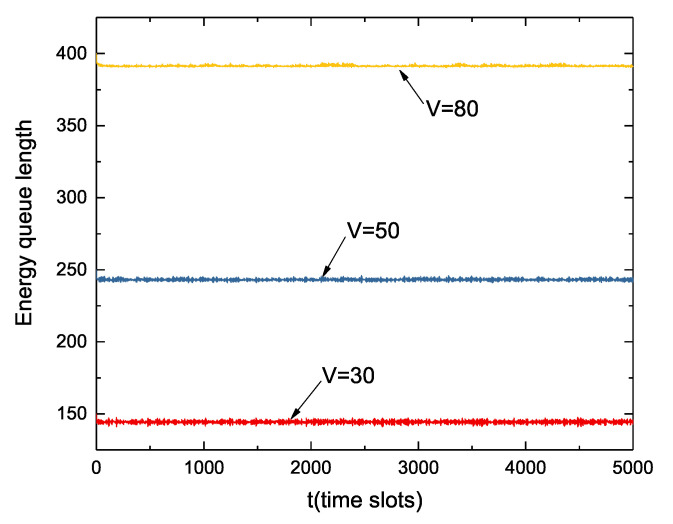
Dynamic change of energy queue under different *V* values.

Figure 5 shows the dynamic change in the length of the energy queue when the values of *V* are different. The length of the energy queue increases as the value of *V* increases. At time slot t=0, the value of the energy queue is equal to the battery capacity, that is, the battery power is full in the initial state. From the figure, we can see that the energy queue is almost like a stable straight line, and the range of fluctuation is relatively stable. This is because nodes transmitting and collecting data consume energy, while the regulation of sleep/wake decisions will properly acquire energy. However, the value of the energy queue will not be greater than the battery capacity.

**Figure 6:**
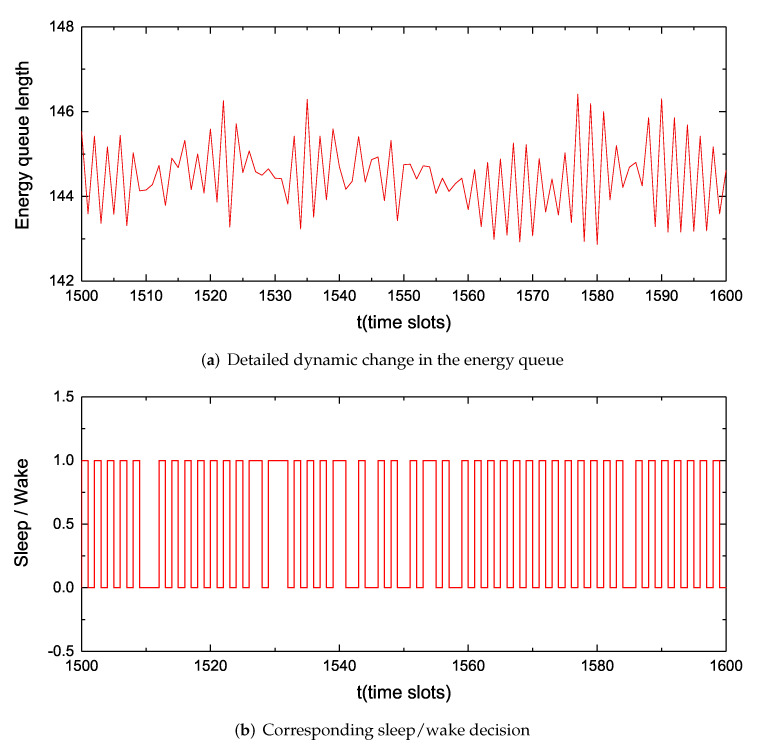
Dynamic change of the energy queue after amplification and corresponding sleep/wake decisions at V=30.

Figure 6a shows the dynamic change of the energy queue with time slots *t* from 1500 to 1600. In the figure, we can clearly see the degree of fluctuation of the energy queue. However, the rise and fall of the energy queue will not continue, because we use the sleep/wake decision to guarantee the energy queue and enhance the sustainable use of electricity. Figure 6b is the sleep/wake decision in correspondence with the energy queue in Figure 6a, wherein 1 represents the wake state and 0 represents the sleep mode. We can roughly see that the proportion of the wake state is about 0.4, which is also the probability that the general node is in the wake state.

**Figure 7:**
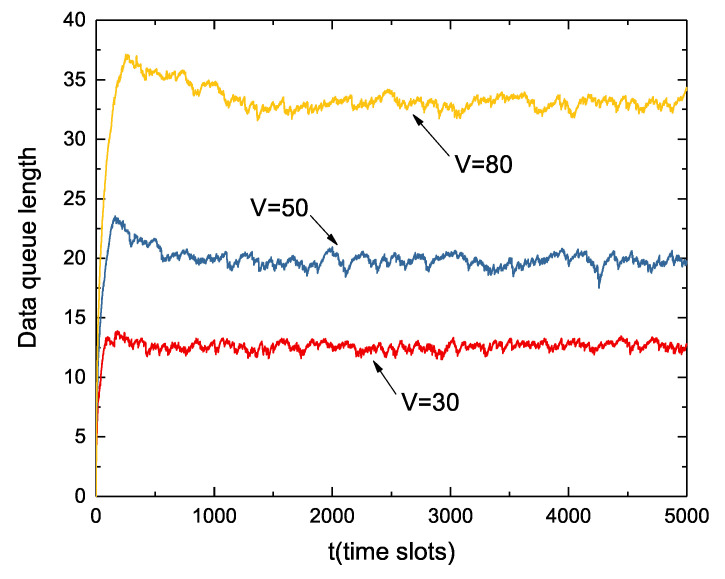
Dynamic change of data queue under different *V* values.

**Figure 8:**
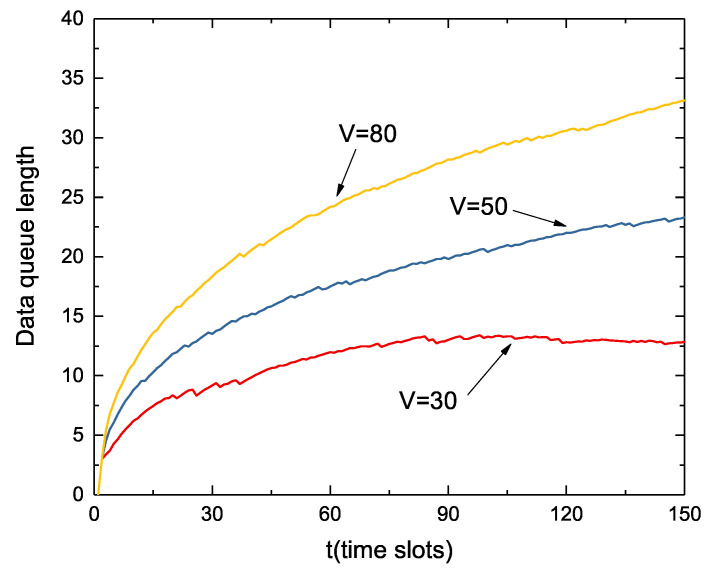
Dynamic change of the data queue after amplification.

Figure 7 shows the dynamic change of the data queue over 5000 time slots at different *V* values. Figure 8 shows the first 150 time slots of the data queue in Figure 7. In the same way as with the energy queue, the length of the data queue also increases as the value of *V* increases. Since the initial state battery is full and the data queue is empty, the node immediately begins collecting large amounts of data. As can be seen from the figure, the growth rate of the data queue decreases as the time slot *t* increases. Although not as stable as the energy queue fluctuation, when the volume of the data queue reaches a certain level, it will remain in a relatively stable range.

### 6.3. Impact of Parameter Changes on Network Utility

**Figure 9:**
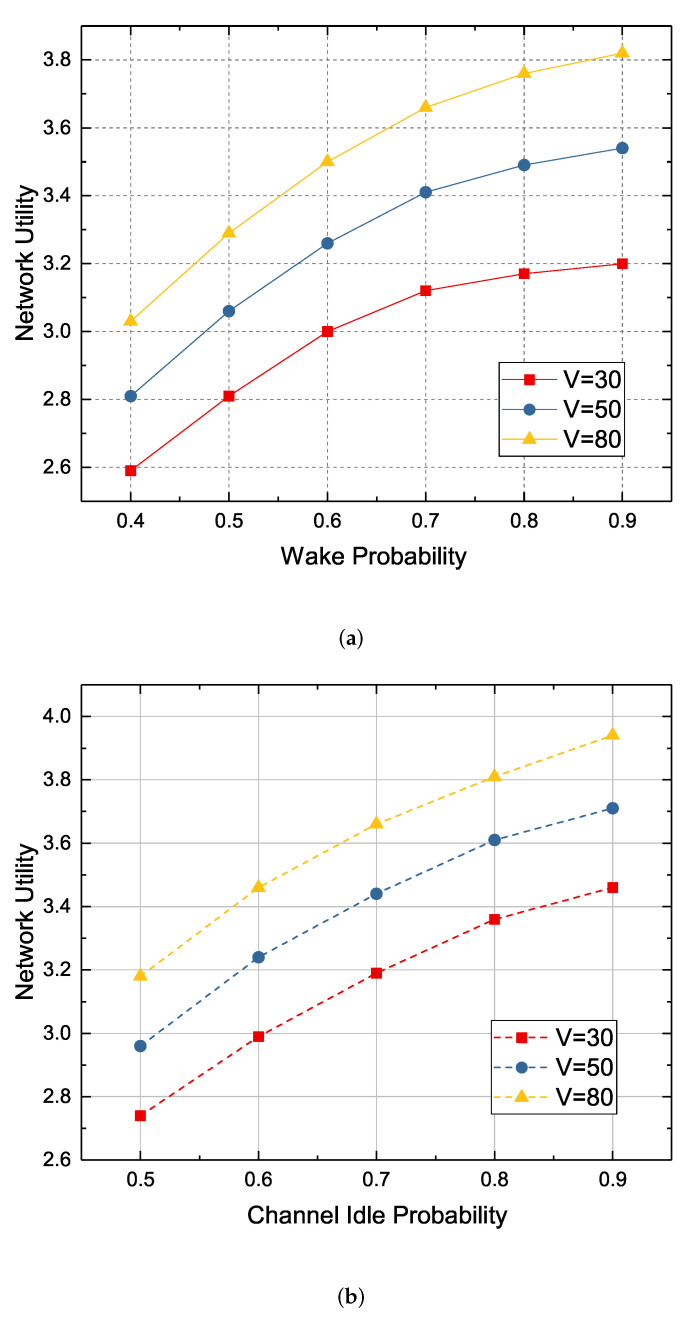
The effect of parameter changes on network utility at different *V* values. (**a**) The relationship between network utility and wake probability; (**b**) The relationship between network utility and channel idle probability.

In this section, we demonstrate the impact of changes of system parameters on network utility. We first examine the impact of wake up probabilities on network utility in sleep/wake decisions. As shown in Figure 9a, as the wake up probability increases from 0.4 to 0.9, the network utility also increases. Yet at the same time, we can find that the growth rate of network utility is declining, because the network utility is also affected by the channel idle probability, the maximum harvestable energy, and the upper limit of energy transmission consumption. Even if the node has a higher probability of being awake, it will be unable to transmit data due to energy shortage, busy channel, and so on.

As shown in Figure 9b, when we increase the channel idle probability from 0.5 to 0.9, the network utility also increases, but the network utility growth rate also decreases. This is also because the network utility is limited by the wake-up probability, the maximum harvestable energy, and the upper limit of energy transmission consumption, that is, the network utility is limited by the influence of multiple system parameters. As shown from both Figure 9a,b, since network utility is limited by multiple parameters, the improvement of network utility by considering only one parameter optimization is limited. The network services cost is also affected by these parameters, therefore, the experimental results of the maximum harvestable energy and the upper limit of energy transmission consumption are used as a comparison of network service cost.

### 6.4. Impact of Parameter Changes on Network Service Cost

**Figure 10:**
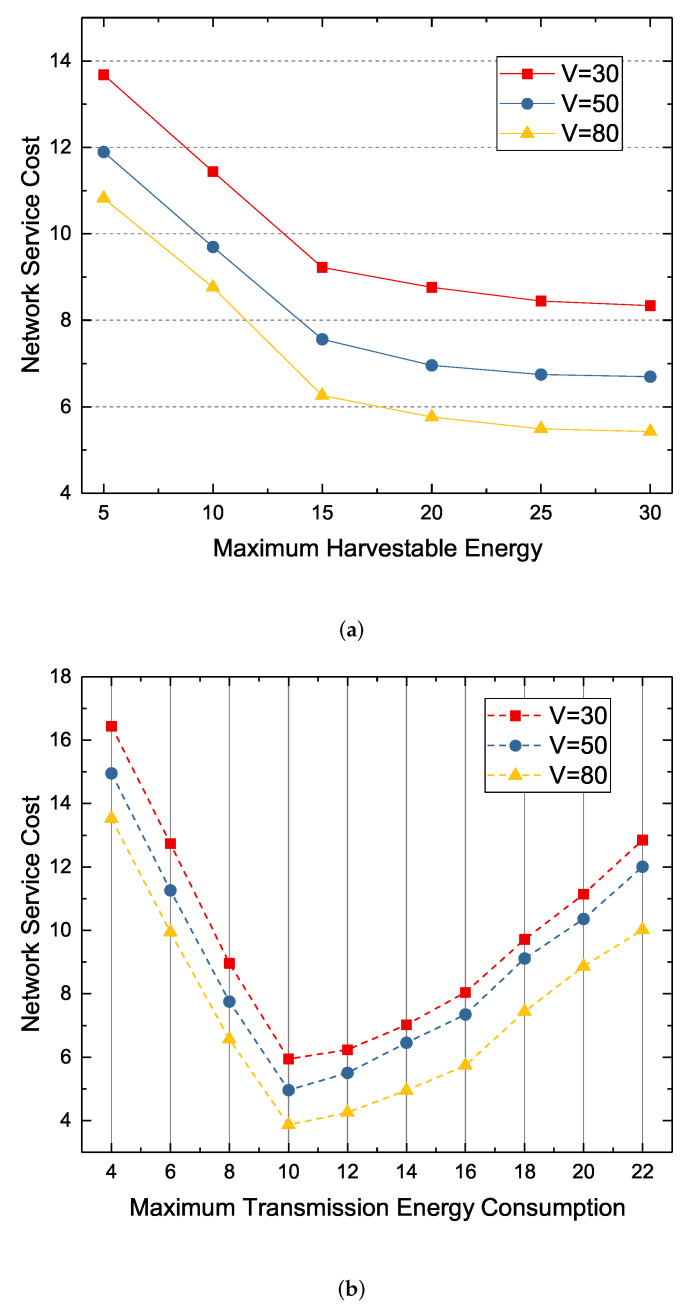
The effect of parameter changes on network service cost at different *V* values. (**a**) The relationship between network service cost and maximum harvestable energy; (**b**) The relationship between network service cost and maximum transmission energy consumption.

In this section, we verify the impact of system parameter changes (primarily, maximum harvestable energy and maximum transmitted energy consumption) on network service cost. We first verify the impact of the maximum harvestable energy on network service cost. As shown in Figure 10a, with the maximum harvestable energy increases, network service cost gradually decreases. This is because nodes have more energy to transfer data, which also results in less redundancy. However, as the maximum harvestable energy increases, the rate of network service cost reduction also decreases because the maximum transmission energy consumption limits the reduction in network service cost in addition to the wake up probability and channel idle probability. Simply put, there is enough energy to transmit data, but the lack of maximum transmission energy consumption is limited to the reduction of network service cost.

As shown in Figure 10b, when we increase the maximum transmission energy consumption from 4 to 22, the network service cost decreases first and then increases. By using a higher maximum transmission energy consumption, the node can transmit more data, that is, the node can fully use the idle channel and make perfect use of the awake state. Therefore, increasing the maximum transmission energy consumption at the beginning will reduce the network service cost, however, when the maximum transmission energy consumption increases later, the node needs to acquire more energy to transmit data. In our experiments, the optimal value for maximum transmission energy consumption is 10, and the value above will increase the network service cost.

## 7. Conclusions

In this paper, we study the joint optimization of data problems and energy problems in WBAN. In order to control the battery to not be too low, we introduce a sleep/wake decision to ensure sufficient power supply. We also propose a new network service cost model to achieve joint optimization of data problems and energy problems by reducing redundancy costs. Specifically, we design an OAA algorithm, which first uses Lyapunov optimization to ensure the stability of the dynamic queue, then decomposes the optimization problem into three sub-problems, and finally achieves the optimal solution one by one, while maximizing network utility and minimizing network service cost. The simulation experiment proves the stability and optimality of WBAN, and also proves the performance of OAA algorithm. We can also verify that the newly introduced network service cost can be significantly optimized.

In OAA algorithm, the most important thing is to balance the relationship among energy harvesting, data transmission, and network occurrence. Our algorithm may not have the strongest energy collection capability, the highest data transmission rate, or the lowest network delay, but our algorithm has the highest energy sustainability and the most stable data transmission, which is most important in WBAN services.

In the future, we plan to continue to use the cost of network services as a measure. In addition, we intend to strengthen the study on the spectrum to ensure channel quality and consider the connection between sensor nodes.

## Figures and Tables

**Table 1:** Simulation parameters and values.

Parameters	Values
ψ	1
ς	1
$P_{G}$	0.2
$G_{max}$	25
$h_{max}$	15
*t*	0.4
$\pi_{s}$	0.5
$P_{max}^{\mu }$	10
$P_{min}^{\mu }$	1
$\beta_{max}$	6
$w_{E}$	1
$w_{Q}$	1
$a_{Q}$	1
$a_{E}$	1

## Data Availability

Not applicable.

## Notations

The following notations are used in this manuscript:

$SW(m_{t})$	Sleep/Wake mode
S(t)	Channel status
G(t)	The amount of data collected by the sensor node in time slot *t*
U(G(t))	Network utility function
β(t)	The channel capacity of the sensor node in time slot *t*
μ(t)	The amount of data transmitted by the sensor node in time slot *t*
Q(t)	The data queue length of the sensor node in time slot *t*
$P_{G}$	The sensor node collects energy consumption per unit of data
$P_{\mu }\left(t\right)$	The energy consumption of data transmitted of the sensor node in
	time slot *t*
$P_{total}\left(t\right)$	The total energy consumption of the sensor node in time slot *t*
h(t)	The harvestable energy of the sensor node in time slot *t*
r(t)	The actual harvest energy of the sensor node in time slot *t*
E(t)	The energy queue length of the sensor node in time slot *t*
N(t)	Network service cost
$a_{E}$	Energy consumption cost per joule
$a_{Q}$	Data transmission cost
f(t)	The utility maximization function of network service cost
	management
$w_{E}$	Weight of energy consumption cost
$w_{Q}$	Weight of data transmission cost

## Appendix A. Proof of Lemma 1

**Proof.** Squaring both sides of Equation (13), and obtaining the system parameter *B*. The details are:

(A1) $$\begin{array}{cc} \frac{1}{2}[Q(t & {+1)}^{2}-Q{\left(t\right)}^{2}]\\ = & \frac{1}{2}\left[{\left[Q\left(t\right)-SW\left(m_{t}\right)\mu \left(t\right)+SW\left(m_{t}\right)G\left(t\right)\right]}^{2}-Q{\left(t\right)}^{2}\right]\\ \leq & \frac{1}{2}{[Q\left(t\right)}^{2}+{\left[SW\left(m_{t}\right)G\left(t\right)\right]}^{2}+{\left[SW\left(m_{t}\right)\mu \left(t\right)\right]}^{2}\\ \; & +2Q\left(t\right)\left[SW\left(m_{t}\right)G\left(t\right)-SW\left(m_{t}\right)\mu \left(t\right)\right]-Q{\left(t\right)}^{2}]\\ \leq & \frac{G_{max}^{2}+\beta_{max}^{2}}{2}+Q\left(t\right)SW\left(m_{t}\right)\left[G\left(t\right)-\mu \left(t\right)\right] \end{array}$$

(A2) $$\begin{array}{cc} \frac{1}{2}[(E\left(t+1\right) & {-O)}^{2}-{\left(E\left(t\right)-O\right)}^{2}]\\ = & \frac{1}{2}[[E\left(t\right)-SW\left(m_{t}\right)P_{total}\left(t\right)\\ \; & {+r\left(t\right)-O]}^{2}-{\left(E\left(t\right)-O\right)}^{2}]\\ \leq & \frac{1}{2}{[r\left(t\right)}^{2}+{\left(SW\left(m_{t}\right)P_{total}\left(t\right)\right)}^{2}\\ \; & +2\left(E\left(t\right)-O\right)\left(r\left(t\right)-SW\left(m_{t}\right)P_{total}\left(t\right)\right)]\\ \leq & \frac{h_{max}^{2}+P_{max}^{2}}{2}\\ \; & +\left(E\left(t\right)-O\right)\left(r\left(t\right)-SW\left(m_{t}\right)P_{total}\left(t\right)\right) \end{array}$$

With the parameters removed, the remainder can be made up of $\Delta_{T,V}\left(t\right)$. The proof is complete. □

## Appendix B. Proof of Theorem 3

**Proof.** We prove Theorem 3 by comparing the OAA algorithm with other random algorithm κ, which uses Lyapunov drift. We use $G^{\kappa }\left(t\right)$, $r^{\kappa }\left(t\right)$, $P_{\mu }^{\kappa }\left(t\right)$ to denote the optimal solution of the random algorithm κ, $\mu^{\kappa }\left(t\right)$ to represent the transmitted data amounts, and $P_{total}^{\kappa }\left(t\right)$ to represent the total energy consumption. In addition, the sleep/wake decision, channel state, and energy acquisition process are independently and equally distributed in each time slot, which is obtained by Theorem 4.5 in [35]:

(A3) $$\left|E\left[\sum_{t=0}^{T-1}\left(G^{\kappa }\left(t\right)-\mu^{\kappa }\left(t\right)\right)\right]\right|\leq \xi_{1}\delta$$

(A4) $$\left|E\left[\sum_{t=0}^{T-1}\left(r^{\kappa }\left(t\right)-SW\left(m_{t}\right)P_{total}^{\kappa }\left(t\right)\right)\right]\right|\leq \xi_{2}\delta$$

wherein δ>0 is an arbitrarily small number, and $\xi_{1}$ and $\xi_{2}$ are normal numbers without any restrictions. In each of the different time slots, OAA algorithm minimizes the Lyapunov drift, which is represented by:

(A5) $$\begin{array}{cc} D_{tot}\left(t\right)=- & \sum_{t=0}^{T-1}\left(\hat{E}\left(t\right)\left(r\left(t\right)-SW\left(m_{t}\right)P_{total}\left(t\right)\right)\right)\\ - & \sum_{t=0}^{T-1}SW\left(m_{t}\right)\left(VU\left(G\left(t\right)\right)-Q\left(t\right)G\left(t\right)\right)\\ - & \sum_{t=0}^{T-1}SW\left(m_{t}\right)Q\left(t\right)\mu \left(t\right) \end{array}$$

Theorem 2 in [36] has been proved, so we can conclude:

(A6) $$\begin{array}{cc} \Delta_{T}\left(t\right)-VE\left[U(G(t))\right]\leq & B+E\left[D_{tot}^{OAA}\left(t\right)|M\left(t\right)\right]\\ \leq & B+E\left[D_{tot}^{\kappa }\left(t\right)\right]\\ \leq & B+\left(\xi_{1}+\xi_{2}\right)\delta -VU^{*} \end{array}$$

where $D_{tot}^{OAA}\left(t\right)$ represents the Lyapunov drift value minimized by OAA algorithm and $D_{tot}^{\kappa }\left(t\right)$ represents the Lyapunov drift value obtained by the κ algorithm. The value of *L* approaches to zero, and we can obtain:

(A7) $$\Delta_{T}\left(t\right)-VE\left[U(G(t))\right]\leq B-VU^{*}$$

We take the expected value of both sides and obtain:

(A8) $$\frac{L(T-1)-L(0)}{T}-\frac{1}{T}E\left[U(G(t))\right]\leq B-VU^{*}$$

L(T−1) and L(0) are finite. We add all the time slots *t* and divide by *T*. Then, we let T→∞ and can obtain $\bar{U}\geq U^{*}-\frac{B}{V}$. The theorem is proved. □
